# The In Vitro Evaluation of Rooster Semen Pellets Frozen with Dimethylacetamide

**DOI:** 10.3390/ani13101603

**Published:** 2023-05-11

**Authors:** Shaimaa K. Hamad, Ahmed M. Elomda, Yanyan Sun, Yunlei Li, Yunhe Zong, Jilan Chen, Ahmed O. Abbas, Farid K. R. Stino, Ali Nazmi, Gamal M. K. Mehaisen

**Affiliations:** 1 Department of Animal Production, Faculty of Agriculture, Cairo University, Giza 12613, Egypt; shaimaa1@vt.edu (S.K.H.); aabbas@kfu.edu.sa (A.O.A.); faridstino@gmail.com (F.K.R.S.); 2 Department of Animal Sciences, The Ohio State University, Columbus, OH 43210, USA; 3 Department of Animal Biotechnology, Animal Production Research Institute, Agriculture Research Center, Giza 12613, Egypt; elomda@vt.edu; 4 Key Laboratory of Animal (Poultry) Genetics Breeding and Reproduction, Ministry of Agriculture and Rural Affairs, Institute of Animal Sciences, Chinese Academy of Agricultural Sciences, Beijing 100193, China; yanyansun2014@163.com (Y.S.); liyunlei@caas.cn (Y.L.); zongyunhe2022@163.com (Y.Z.); chen.jilan@163.com (J.C.); 5 Department of Animal and Fish Production, College of Agricultural and Food Sciences, King Faisal University, Al-Ahsa 31982, Saudi Arabia; 6 Food for Health Discovery Theme, The Ohio State University, Columbus, OH 43013, USA

**Keywords:** cryopreservation, roosters, semen, dimethylacetamide, sperm motility

## Abstract

**Simple Summary:**

Semen cryopreservation remains the most applicable methodology for conserving and transmitting superior genetic backgrounds in poultry species. However, there is wide variability in the results of sperm viability and fertility due to susceptibility to cryodamage. The current study implemented a cryopreservation protocol based on rapid freezing of semen pellets supplemented with various levels of dimethylacetamide (DMA), focusing on in vitro quality, computer-assisted-sperm-analysis (CASA), and anti-freeze-associated gene expression of post-thawed rooster sperm. The current investigation provides novel results regarding the appropriate concentration and molecular mechanism by which DMA could be supplemented to frozen semen pellets and used as a successful protocol for sperm cryopreservation in poultry species.

**Abstract:**

Sperm cryopreservation is an effective technique for conserving animal genetic diversity and transmitting superior genetic backgrounds, maintained via a non-invasive sampling and collection of huge quantities of sperm. Nevertheless, cryopreservation in avian species is not commercially viable because of the rooster sperm’s susceptibility to damage. This study aims to estimate the impact of dimethylacetamide (DMA) as a cryoprotectant at different levels (3%, 6%, or 9%) on the post-thawed sperm quality, motility, antioxidant-biomarkers, and the expression of anti-freeze related genes. Semen samples were collected twice a week from twelve roosters aged 40 wk, weighing 3400 ± 70 g, and belonging to the Cairo-B2 chicken strain. Fresh semen samples were rapidly appraised, pooled, diluted with two volumes of a basic extender, and divided equally into three groups. The diluted groups were chilled at −20 °C for 7 min, then gently supplemented with 3, 6, or 9% pre-cooled DMA and equilibrated at 5 °C for a further 10 min. Semen pellets were formed by pipetting drops 7 cm above liquid nitrogen (LN_2_), which were then kept inside cryovials in the LN_2_. Thawing was performed 2 months later by taking 3–4 pellets of the frozen semen into a glass tube and warming it in a water bath for 8 s at 60 °C. The results showed that 3% DMA increased the proportion of total motile sperm, progressivity, viability, and plasma membrane integrity (%) compared to the 6% and 9% DMA groups. The lipid peroxidation and antioxidant enzyme activity were improved in the 3% group. At the same time, some anti-freeze-related genes’ (including ras homolog family member A (RHOA), heat shock protein 70 (HSP70), and small nuclear ribonucleoprotein polypeptide A (SNRPA1)) expressions were upregulated within the 3% DMA group relative to other groups. In conclusion, the 3% DMA group maintained higher post-thawed sperm quality than the other tested groups.

## 1. Introduction

Sperm cryopreservation is critical to preserve avian species’ genetic diversity, specifically with high-value species. Nonetheless, the cryopreservation technology of poultry sperms is very limited in the poultry industry because of the rooster sperm sensitivity to freezing [1]. Unlike mammals, rooster spermatozoa have unique characteristics that may be the reason for the unsuccessful cryopreservation of chicken semen. Rooster spermatozoa have smaller cytoplasm contents, antioxidants, and mitochondria and higher polyunsaturated fatty acid levels within the plasma membrane than their counterpart in mammals. Thus, rooster spermatozoa are more susceptible to damage throughout the freezing-thawing process [1,2].

The cryoprotectant (CPA) and the freezing protocol are the most important factors in the cryopreservation process. Glycerol (GLY), dimethylacetamide (DMA), ethylene glycol (EG), and dimethyl sulfoxide (DMSO) are the most commonly studied cryoprotectants [3,4]. However, these CPAs (GLY, DMA, and DMSO) are toxic for poultry sperms. GLY was known to cause the least toxicity but has contraceptive activity [5]. Thus, GLY removal is required before artificial insemination by adding an extender and centrifugation [4]. With DMA, however, there is no need to remove it from the frozen/thawed sperm since no contraceptive effect has been proven [6], and subsequently, less sperm damage and better sperm fertility were detected.

Moreover, there are different types of sperm packaging and freezing protocols [3] that have been tested for their efficiency in protecting sperm from the damage caused by freezing in avian species. Semen is stored during the cryopreservation process as pellets or in plastic straws. The freezing protocol and packaging method affect the semen cryopreservation process outcome [7]. Vitrification, which forms the pellets during cryopreservation, is a process of rapid cooling of a liquid solution that makes the viscosity so large and arrests the molecular diffusion without ice crystal formation. At this stage, the sample is called a glass or vitreous solid [8]. The osmotic damage effect occurs during thawing and varies according to the thawing temperature. To avoid ice crystal formation, high warming rates are needed during the warming process [9].

Semen has a strong antioxidant system that contains superoxide dismutase (SOD), catalase (CAT), and glutathione peroxidase (GPX) [10,11]. Semen antioxidant enzymes have been investigated in humans [12] and pigs [13]. This antioxidant system facilitates adequate sperm protection. Additionally, the antioxidant system functions similarly in the bird spermatozoa, such as chickens, geese, turkeys, guineas, and fowlswork [10,11].

Antioxidant enzyme activity is influenced by cryopreservation, which induces the intensity of lipid peroxidation (LPO) [10]. Thomson, et al. [14] reported that sperm damage mechanisms affect post-thaw sperm viability, motility, acrosome, and plasma membrane integrity. In the sperm plasma membrane, during high oxidative stress and LPO, the antioxidant enzyme activities are induced because the enzymes are fully utilized for sperm protection [10].

Partyka and Niżański [15] stated that semen storage procedures induce higher levels of malondialdehyde (MDA) and increase osmotic stress, which causes oxidative stress in semen. Moreover, the avian sperm plasma membrane has a high polyunsaturated fatty acid level, making it more liable for oxidative damage during the freezing-thawing procedures [16]. Therefore, peroxidation in the frozen-thawed sperm is more likely to occur compared to the fresh sperm [17]. In addition, Izanloo, Soleimanzadeh, Bucak, Imani and Zhandi [16] showed that semen quality decreased with the higher lipid peroxidation levels during the freezing-thawing process.

The current research is dedicated to exploring the impact of DMA concentration on the post-thawed semen that was stored as pellets. Different parameters were investigated, including post-thawed sperm acrosome integrity, viability, motility, and membrane status in roosters. Moreover, the antioxidant defense status and the expression of the anti-freeze-related genes have been investigated in the post-thawed sperm.

## 2. Materials and Methods

### 2.1. Animals and Ethical Statement

A total of twelve roosters of Cairo-B2 chicken strain (40 weeks old, 3400 ± 70 g weight, 18th generation) were used for the current study. The Cairo-B2 chicken strain was established in the Animal Production Department (Faculty of Agriculture, Cairo University, Egypt) by crossing between males from Arbor Acers grandparent-female-line with females from White Baladi native Egyptian chicken breed, and selecting the next generation of the parents based on the highest live body weight at 42 d of age [18]. The chickens were reared in cages (50 × 50 × 60 cm) and held in the Agricultural Experiments Station (Cairo University). All birds have been maintained under similar environmental circumstances (16L:8D photoperiod, 20–25 °C, and unlimited access to water). A typical commercial diet was used (2750 Kcal metabolizable energy and 14% crude protein), and it was manufactured by Feedmix-Egypt Co., Obour City, Kaliobeya, Egypt.

The protocols adopted in this study have been approved and accomplished following the Institutional Animal Care and Use Committee (CU-IACUC) policies at Cairo University (Approval no. CU-II-F-12-20).

### 2.2. Semen Collection and Freezing/Thawing Protocol

The roosters were trained for semen collection and routinely used as sperm donors since puberty (24 weeks of age). The semen samples were collected two times per week from each male by the same operator for as long as 4 consecutive weeks using the abdominal massage technique and then the cloacal stroking [19]. The samples were collected in sterilized glass tubes, which were placed in a water bath at 37 °C. Sperm motility was measured by the computer-assisted sperm analysis (CASA; SpermVision™ software, version 3.0 for Win10, Minitube, Tiefenbach, Germany). The non-contaminated samples with approximately 4 × 10^9^ sperm/mL and 60% progressive motility were chosen and pooled (only six semen pools were considered in this study). Then, the samples were diluted (1:2 (*v*/*v*)) with a basic EK extender provided by Lukaszewicz [20] and evenly allocated into three groups according to the DMA concentration. The diluted groups were first subjected to rapid cooling for 7 min at −20 °C. Then, the pre-cooled DMA was added slowly to these groups with concentrations of 3, 6, or 9% and left for 10 min for equilibration at 5 °C. To form pellets, semen drops of approximately 70 μL were pipetted from 7 cm above liquid nitrogen (LN2), then transferred into cryovials, and kept inside the LN2. Two months later, 3–4 pellets of the frozen semen were taken into a glass tube and thawed by putting it within a water bath for 8 s at 60 °C.

### 2.3. Sperm Motion Characteristics

Sperm motility was assessed for the thawed samples by putting a semen drop on a prewarmed (37 °C) glass slide using CASA. Approximately 2500 cells were randomly captured and analyzed by the CASA system for each sample. The sperm motion parameters, which include total motility (TMOT, %), progressive motility (PROG, %), average path velocity (VAP, µm/s), straight-line velocity (VSL, µm/s), curvilinear line velocity (VCL, µm/s), linearity (LIN = VSL/VCL%), straightness (STR = VSL/VAP%), wobble (WOB = VAP/VCL%), the amplitude of lateral head displacement (ALH, µm), and beat cross frequency (BCF, Hz).

### 2.4. Sperm Quality Characteristics

#### 2.4.1. Viability

The viability of sperm (% Live/Total sperm) of each group was examined utilizing an Eosin-Nigrosin stain, following Murugesan and Mahapatra [21] with slight modification. In brief, 10 μL of 25% eosin-nigrosin staining solution (Bio-Diagnostic, Inc., Giza, Egypt) was added to 10 μL of the thawed semen, followed by incubation for 30 s at room temperature before dispersing on a microscopic glass slide and air drying. Then, about 200 sperm were examined using a phase-contrast microscope with a 1000× magnification maintained via oil immersion. Live spermatozoa showed as unstained, while dead spermatozoa showed as pink-stained.

#### 2.4.2. Acrosome Integrity

The percent of sperm cells with intact acrosome was estimated using phase contrast microscopy, by counting 200 Giemsa blue-stained cells under a 1000× magnification, following Rakha, et al. [22] with a few amendments. Giemsa staining involved dispersing 20 μL of thawed semen over a glass slide, permitting them to dry, and fixing them in a fixative solution (100 mL formalin 35%, 9 g sodium chloride, and 12 g dibasic/anhydrous Na_2_HPO_4_ dissolved in 900 mL distilled water) for 15 min. Fixed slides were kept in Giemsa stain (Bio-Diagnostic) diluted with distilled water (1:4 *v*/*v*) for 90 min, cleaned via distilled water, and left to dry in air. A total of 200 sperm cells were examined in four distinct fields at least. To compute the acrosome-intact sperm percentage, sperm cells having blue-tainted acrosomal caps were distinguished from those untainted within each sample. All semen samples were analyzed by one laboratory tech individual.

#### 2.4.3. Plasma Membrane Status

Hypoosmotic swelling test (HOST) was utilized in assessing the sperm membrane functionality depending on the method given via Rakha et al. [22] with a modification. HOST allowed selecting criteria like curled and swollen tails. Adding aliquots of 10 μL of thawed sperm mixed with 100 μL of prewarmed swelling solution (1.375 g fructose and 0.75 g sodium citrate dihydrate within 100 mL distilled water, 100 mOsmol/kg) was utilized in estimating the sperm membrane integrity. After incubating samples for one hour at 37 °C, 20 μL of the resulting mixture was dispersed on a glass slide and fixed within formal saline for 15 min. A phase contrast microscope (1000× with oil immersion) was utilized to examine 200 sperm cells. Swollen or coiled tails represent the key signals for recognizing perfect plasma membrane functionality, denoted by the percentage of spermatozoa with intact plasma membranes.

### 2.5. Sperm Antioxidant Defense Status

#### 2.5.1. Semen Extraction

Six replicates were used for each treatment. Each replicate was formed by thawing 8 pellets of frozen semen and was approximately 0.5 mL. About 0.5 mL of phosphate buffer saline (PBS, pH 7.4) was provided to each replicate (≈400 × 10^6^ sperm/mL per sample). PBS was used to wash the samples twice, then placed in a centrifuge operated at 1030× *g* for 10 min at 4 °C to collect precipitates. The pellets were resuspended in 1 mL of PBS comprising 4% Triton X-100, incubated for 30 min at room temperature, and then placed in a centrifuge operated at 1030× *g* for 20 min at 4 °C. The supernatant has been kept at −20 °C for additional analyses.

Total protein was measured to normalize the obtained data according to the Biuret reaction method [23], following the manufacturer’s procedure of colorimetric assay kit (TP-2020, Bio-Diagnostic). Briefly, 25 μL of the standard solution or the sample was incubated with 1 mL Biuret reagent for 10 min at 37 °C. Sample absorbance (Asample) and standard (Astandard) were reported against a reagent blank at 550 nm through a scanning spectrophotometer (CE1010, Cecil Instruments Limited, Cambridge, UK). The formula used to calculate protein concentration (g/dL) was (Asample/Astandard × 5).

#### 2.5.2. Total Antioxidant Capacity (TAC)

The total antioxidant status of the semen sample was measured by TAC colorimetric kit (TAC-2513, Bio-Diagnostic) following the previously described methods [24]. According to the kit’s protocol, the TAC was assessed by adding 20 μL of the sample to 500 μL of H₂O₂ substrate and incubating at 37 °C for 10 min, followed by incubating the mixture with 500 μL of working chromogen reagent at 37 °C for 5 min. The sample (AS) and blank (AB) absorbance were directly reported against distilled water at 505 nm. TAC concentration, which is presented as μM/mg protein, was computed (AB − AS × 3.3).

#### 2.5.3. Superoxide Dismutase Activity

A colorimetric assay kit (SOD-2521, BioDiagnostic, Inc., Giza, Egypt) was used to evaluate the SOD activity. The nitro blue tetrazolium (NBT) reduction method was used to measure SOD activity [25]. Briefly, 1 mL of working reagent (1 mL NADH, 1 mL NBT, and 10 mL phosphate buffer pH 8.5) was added and mixed well to 100 μL of the control sample (distilled water), then 100 μL of phenazine methosulphate was added to initiate the reaction. The elevation of absorbance at 560 nm over 5 min for the sample (ΔAsample) and the control (ΔAcontrol) was estimated at 25 °C. The SOD activity was computed as unit/assay [U = (ΔAcontrol − ΔAsample)/ΔAcontrol × 100 × 3.75] and subjected to further normalization per mg protein within every sample.

#### 2.5.4. Glutathione Peroxidase Activity

The GPX estimation was achieved by NADPH and oxidized glutathione. Then, the oxidized glutathione was reduced via GPX with a simultaneous NADPH oxidation to NADP+. The GPx activity was measured depending on the procedures described by Koracevic et al. [24]. Based on the ‘kits’ approach (GPx-2524; BioDiagnostic, Inc., Egypt), 10 μL of the sample was mixed with a reaction mix comprising 100 μL of reagent (glutathione reductase, glutathione, and NADPH), 1 mL of assay buffer (phosphate buffer and Triton X-100, pH 7.0), and 100 μL of hydrogen peroxide (diluted 100 times). The absorbance reduction at 340 nm per minute (ΔA340) was reported against deionized water over 3 min. An adequate diluting was utilized to control the beginning of A340 at 1.5 and ΔA340 at 0.05 per minute. The GPX activity findings were reported as units of GPX (ΔA340/0.00622 × dilution factor) and subjected to further normalization per mg protein within every sample.

#### 2.5.5. Lipid Peroxidation

The MDA analysis is an indicator of the lipid peroxidation level. Thus, MDA was analyzed using the colorimetric assay kits (MDA-2529, Bio-Diagnostic) and following the procedures reported by Kei [26]. Kei’s method indicated heating 200 μL of the sample or standard in a boiling water bath for 30 min with 1 mL of chromogen. After cooling, the sample absorbance (Asample) was estimated against the blank, while the standard absorbance (Astandard) was estimated at 534 nm against distilled water. The calculation of MDA level was performed using the formula (Asample/Astandard × 10) and was presented as micromoles of MDA per sperm concentration.

### 2.6. Anti-Freeze Related Genes Expression

Preceding total RNA extraction, semen samples were subjected to the purification process. A total of three replicates per treatment (8 pellets × 70 µL of frozen/thawed semen) and control (450 µL of fresh semen) groups were cleansed from immature spermatocytes and somatic cells as given by. Briefly, each replicate was supplemented with about 0.5 mL of PBS. PBS was used to wash the samples twice and centrifuged for 10 min at 1030× *g* at 4 °C to gather precipitates. The resultant pellets were re-suspended with 1 mL PBS and encrusted over 3 mL of prewarmed (37 °C) density gradient medium (Density: 1.077 g/mL, 40% Pancoll human, PAN-Biotech GmbH, Aidenbach, Germany). Then, samples were centrifuged for 30 min at 300× *g* within room temperature. The bottommost layer was cautiously transported into a 2 mL cryovial, utilizing a Pasteur pipette, and rewashed two times via PBS.

Total RNA was removed from the refined sperm specimens of fresh and frozen/thawed semen utilizing the GeneJET™ RNA Purification Kit (Thermo Fisher Scientific, Waltham, MA, USA). All the extraction steps were performed according to the manufacturer’s guidelines. Afterward, cDNA synthesis succeeded by qPCR assay utilizing SuperScriptTM III One-Step RT-PCR System with Platinum^®^ Taq DNA Polymerase kit (Invitrogen, Thermo Fisher Scientific). Quantitative real-time polymerase chain reaction (qRT-PCR) assays were run for four target anti-freeze-related genes [27]. The Primer-BLAST web tool was used to design the primers for the four designated genes, as listed in Table 1. To normalize the analytical differences of target genes, the chicken glyceraldehyde 3-phosphate dehydrogenase (GAPDH) was utilized as the reference gene. Fold variation was estimated using the 2^−ΔΔCt^ relative quantification technique reported by Livak and Schmittgen [28].

### 2.7. Statistical Analysis

SPSS 22.0 (IBM corp., New York, NY, USA, 2013) was used for statistical analysis. Six pools from each treatment group were used (*n* = 6) and ascribed as investigational units for all parameters of sperm motion, quality attributes, and antioxidant defense status. In contrast, three pools (*n* = 3) from each treatment group were ascribed as investigational units to assess the anti-freeze gene expressions. The statistical differences between different DMA concentrations (3%, 6%, and 9%) during freezing on all estimated variables were analyzed by one-way analysis of variance (ANOVA) followed by the Tukey post hoc test. Data were assessed for normality using the Shapiro–Wilk test before the ANOVA. The Box-cox transformation function was applied for the non-normal distributed data. The results are displayed after back transformation as mean ± standard error (SE), and the significance was adopted at a *p* value of less than 0.05.

## 3. Results

### 3.1. Sperm Motion Characteristics

The 3% DMA group reveals significantly (*p* < 0.05) elevated percentages of PROG motility relative to the 6% and 9% DMA groups. Similarly, the TMOT of the 3% DMA group was significantly (*p* < 0.05) higher relative to the 9% DMA group (Table 2). However, no significant variances were observed regarding the VSL, VAP, VCL, LIN, STR, ALH, WOB, or BCF between the different groups of DMA concentrations (*p* > 0.05).

### 3.2. Sperm Quality Characteristics

As shown in Table 3, the 3% DMA group revealed higher sperm quality parameters than the other groups (*p* < 0.05). In addition, the sperm viability and acrosome integrity decreased when semen was frozen in the case of 9% DMA relative to the lowered DMA concentrations. In addition, the plasma membrane integrity of the frozen sperm significantly (*p* < 0.05) decreased when using concentrations higher than 3% DMA.

### 3.3. Sperm Antioxidant Defense Status

The TAC of post-thawed semen had not been affected by variable DMA concentrations. The GPX activity revealed a significant (*p* < 0.05) decrease in the 9% group (0.66 mU/mg) compared to the 3% MDA group (2.12 mU/mg), and no significant differences in the 6% group (1.39 mU/mg) were observed. Additionally, the SOD activity was significantly decreased in the 6% and 9 % groups (15.92 and 14.52 U/mg) compared to the 3% MDA group (23.57 U/mg). A significantly elevated MDA level was found within the frozen semen with high concentrations of MDA (6% and 9% groups) (0.111 and 0.114 nM/mg) compared to the 3% MDA group (0.054 nM/mg) (Table 4).

### 3.4. Anti-Freeze Related Genes Expression

**Figure 1:**
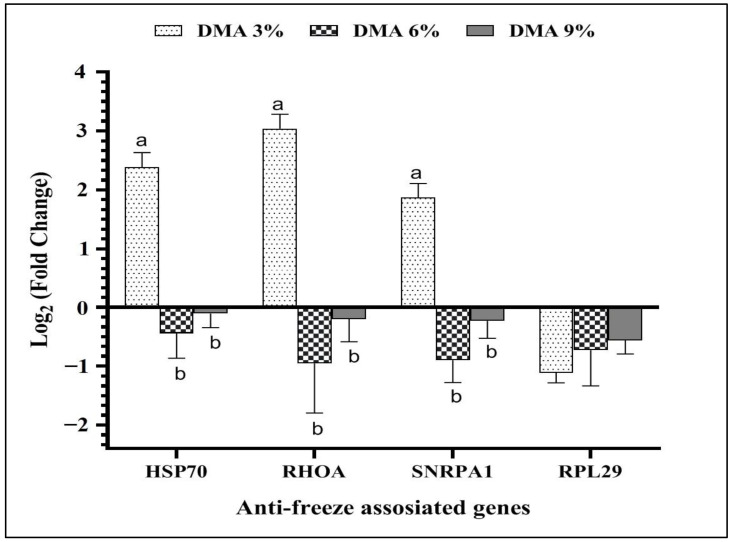
Impact of different DMA concentrations in freezing extender on the anti-freeze-related genes expression of post-thawed rooster sperm. Bars demonstrate the means of 3 samples (*n* = 3) as log_2_ (fold change) ± standard error (SE). ^a,b^ Within the same gene, means denoted by various superscripts are significantly different (*p* < 0.05). HSP70: heat shock protein family A, RHOA: ras homolog family member A, SNRPA1: small nuclear ribonucleoprotein polypeptide A’, and RPL29: ribosomal protein L29.

Figure 1 illustrates the findings of anti-freeze-related genes of rooster sperm frozen with DMA. The HSP70, RHOA, and SNRPA1 genes were significantly (*p* < 0.05) upregulated in the 3% DMA group relative to the 6% and 9% DMA groups. DMA concentrations revealed no considerable impact in post-thawed rooster sperm regarding the RPL29 gene expression.

## 4. Discussion

Gamete cryopreservation has been used to preserve animal genetic resources in several species over the last 65 years. Some factors control the success of the cryopreservation process in avian species, including the packing method (straws or pellets) and freezing/thawing rates that induce the readjustment of sperm’s function and structure [6].

Decreasing the temperature during cooling/freezing processes causes cell biological damage due to promoting the cells to adjust to the osmotic and thermic changes [5,29]. However, this cell damage can be either reversible or permanent. Reversible damage can be reported as a temporary injury of the structure or malfunction of the membrane permeability, while permanent damage can be detected by lack of motility [30]. Hence, intracellular CPAs became important to protect the cells from the temperature ‘fluctuations’ effects and minimize the damage caused by the ice crystals [5,30]. However, if used at high concentrations, the CPA can be harmful to the cells [5,30]. Therefore, it is critical to balance the positive and the negative effects of the CPA.

The current study showed that the 3% DMA group exhibits significantly (*p* < 0.05) higher proportions of TMOT and PROG motility compared to the 6% and 9% DMA groups. This result is supported by previous research which reported higher sperm motility at 3% DMA than the other treatments in local Egyptian strains (Dokki-4 and El-salam) [31] and Mediterranean chicken breeds [7], as well as in the Birchen Leonesa breed and wild birds [32]. Abouelezz et al. [7] reported that the native Mediterranean chicken sperm is sensitive to DMA, and concentrations lower than 6% are recommended, while O’Brien et al. [32] stated that the EK media improved the cryopreservation results with 4% DMA levels compared to the lower levels. On the other hand, DMA addition at the concentration of 6% can retain sperm motility and fertility after cryopreservation in chicken [33], 8% DMA in turkey [34], and red jungle fowl [6]. In turkeys, Iaffaldano et al. [34] stated that high levels of DMA showed better cryoprotection during the freezing process for the semen pellets. It is suggested that the high concentrations of permeable cryoprotectants are responsible for high osmolality in the extender. In addition, CPAs are required to maintain cell dehydration and subsequently minimize the damage caused by ice crystal formation during fast freezing. The freeze/thaw process optimization requires the balance between all the factors involved to minimize the intracellular ice crystal formation and the exposure to high solutes, which cause cell injuries [33].

In this study, the highest sperm viability was recorded in the 3% DMA group. This result agrees with Abouelezz et al. [7] and Roushdy et al. [31]. They indicated that the lower concentration of DMA at 3% improved the viability results compared to the high concentration. However, previous studies showed that the highest sperm viability was recorded in the 6% DMA group [32,35]. Castillo et al. [29] documented the absence DMA concentration effect on the viability, whereas Annelisse et al. [35] reported that the highest viability was recorded with 6% DMA. On the other hand, sperm viability in chickens was negatively affected by the low DMA concentration [36], while the high CPA concentration induced higher proportions of intact cell membranes [33].

The present study shows that the post-thawed semen group with low sperm motility had a significantly higher MDA concentration than those with high sperm motility. This finding indicates the occurrence of higher oxidative stress in the lowered sperm motility group compared to the group with high sperm motility. These results show agreement with previous studies on fresh chicken semen [10,37,38]. Mussa et al. [38] reported that the increase in MDA content caused a decrease in sperm quality, especially sperm motility. Moreover, the increase in lipid peroxidation promotes sperm oxidative stress, subsequently decreasing sperm motility in avian species [10]. Partyka, et al. [39] reported that frozen/thawed avian semen might have a weaker antioxidant system compared to fresh semen, and subsequently, frozen/thawed semen is more susceptible to lipid peroxidation. Additionally, Ansari, et al. [40] stated that in Indian Red Jungle Fowl, the freezing/thawing strategy increased the MDA concentration and decreased the total antioxidant capacity. In addition, the sperm membrane lipid bilayer is negatively affected by lipid peroxidation by changing its permeability and fluidity and altering cell integrity [38]. These subsequent events induced by the presence of a high MDA content in the frozen sperm cells can explain the low sperm motility after thawing.

The current study reveals significant (*p* < 0.05) elevation in the activity of GPX and SOD of the 3% DMA group compared with the 9% DMA group, while the MDA levels were significantly lower than the 6% and 9% DMA groups. Thus, this high antioxidant enzyme activity of GPX and SOD might change the response to the altered MDA concentration and sperm motility to protect the sperms from the effect of ROS and lipid peroxidation to maintain high sperm quality, especially motility. Mussa et al. [38] agreed with these results. They reported that the antioxidant enzyme activity could change in response to the MDA concentration and sperm motility to avoid the consequences of ROS and lipid peroxidation on the quality of sperm. Our study indicates an inverse correlation between antioxidant enzymes (GPX and SOD) and MDA.

On the contrary, Mavi, Dubey and Cheema [37] reported a positive correlation between lipid peroxidation and antioxidant enzyme activity. In addition, Partyka et al. [11] reported a negative correlation between GPX activity and integrity of the sperm plasma membrane. Moreover, Kasimanickam, et al. [41] reported a negative correlation between the GPX activity and the proportion of sperm progressive motility in rams. Additionally, there was a greater activity of GPX with the group that showed lower semen quality. Additionally, Partyka, Łukaszewicz and Niżański [11] stated that frozen/thawed semen with the highest viability had a higher SOD activity than the other groups in boar.

To the extent that we know, the current study is among the first contributions in investigating the expression of the anti-freeze-related genes in the DMA-frozen sperm of chickens. Singh et al. [42] explored the transcript profile in chicken sperm using a microarray technique and reported the existence of thousands of encoded transcripts. Moreover, Qi et al. [27] reported the downregulation in 2086 genes and the upregulation in 29 genes expressed in the frozen/thawed semen compared to fresh sperm in roosters using the microarray technique. In addition, Qi et al. [27] stated that the sperm fertilizing ability could be stimulated by gene expression modification for the genes responsible for cellular, molecular, and biological functions during the freezing/thawing process. For instance, a member of the heat shock protein family called HSP70 has been linked to the control of the development and function of the reproductive system and has become a potent predictive biomarker of male fertility [43]. Additionally, Wang, et al. [44] stated that HSP90 was found in the sperm tail and has a role in sperm fertility. Thus, HSP70 and HSP90 expression showed downregulation after freezing/thawing application on chicken sperm [27]. Similarly, HSPA8, a highly conserved subtype of HSP70, considerably contributes to rooster sperm cryopreservation [45].

In the current investigation, the sperm motility, the integrity of the plasma membrane, and the HSP70 expression were increased after the freezing/thawing procedure within the 3% DMA group. The results suggested that HSP70 expression might be the reason for increasing sperm motility and plasma membrane integrity. Zhang, et al. [46] support these outcomes, recognizing that HSP70 expression was upregulated in the sperm cells which have higher motility and integral plasma membranes, and vice versa. Moreover, Shrum, et al. [47] stated that the deletion of the HSP70 promoter affected sperm motility and velocity in bulls. In addition, HSP70 was reported to increase sperm longevity and viability in boar and bull [48] and regulate cell function through enzyme activity [46].

In our study, the HSP70 was upregulated along with the increase in sperm motility, which may indicate that the change of HSP70 expression caused an increase in the antioxidant enzyme activity, a decrease in ROS, and subsequently, an increase in sperm motility. On that principle, HSP70 may have a cryoprotective role similar to HSP90 in protecting sperm cells against oxidative stress [49] and/or decreasing intracellular energy consumption [50]. These results agreed with other studies suggesting that HSP90 collaborates with HSP70 and may function against oxidative stress [50]. In addition, it was reported that HSP70 expression levels increased the SOD activity and protected sperm membranes during stress [46]. Zhang et al. [46] found a positive correlation between HSP70 expression and motility in bull sperm and suggested that HSP70 suppressed the ATP degradation, thus providing sperm with enough energy to express additional HSP70 and keep moving after the freezing/thawing process.

RhoA is a small guanosine triphosphatase protein [51], which functionally participates in cytoskeleton regulation, chromosome inheritance control, and cellular motility and adhesion [52]. Chen, et al. [53] reported that RhoA considerably modulates actin polymerization and alters cell membrane stability; thus, RhoA likely contributes to the sperm capacitation process. Our results indicate that RHOA expression was upregulated in the 3% DMA group, as well as the sperm quality results being improved within the same group in comparison with the 6% and 9% DMA groups. These outcomes disagreed with Gu et al. [52], who reported substantial downregulation of the *Rhoa* gene expression during the freezing/thawing process in murine semen. Moreover, Qi et al. [27] stated that the *Rhoa* expression with the freezing/thawing process was significantly downregulated in rooster semen.

Small nuclear ribonucleoprotein polypeptide A-prime (SNRPA1) is among the specific protein elements of the U2 snRNP (small nuclear ribonucleoprotein) particle, and it regulates sperm maturation [54]. Like HSP70 and RHOA genes, our results indicate that SNRPA1 expression was markedly upregulated in the 3% DMA group relative to the elevated levels (6% and 9%). The upregulation of SNRPA1 expression is probably owed to the increased sperm motility within the group of 3% DMA, since the lack of SNRPA1 causes an accumulation of mitotic spermatogonia that fail to differentiate into spermatocytes and mature sperm, as reported by Wu et al. [54].

RPL29, as a ribosomal protein, might be a critical factor in fertilization and/or spermatogenesis [55]. Qi et al. [27] reported that RPL29 expression in rooster sperm was substantially downregulated following cryopreservation. However, our study did not show any significant effect on the RPL29 expression using various levels of DMA in post-thawed sperm, and these results are in agreement with other studies [2]. On the other hand, Riesco and Robles [56] reported that cryopreservation of Zebrafish genital ridges downregulated most of the gene transcripts and demonstrated that this effect was a result of the freezing/thawing procedure rather than exposure to CPAs themselves. Therefore, additional molecular research is required to investigate the effect of CPAs on avian sperm cells during the cryopreservation process.

## 5. Conclusions

In the current study, the impact of freezing rooster semen pellets with different DMA concentrations on the post-thawed sperm motility, viability, acrosome integrity, and plasma membrane status, as well as antioxidant defense and anti-freeze gene expression were investigated. The 3% DMA concentration in the freezing semen extender results in an increased percentage of total and progressive motile sperm of viable sperm with intact acrosomes and plasma membranes in comparison with 6% and 9% DMA concentrations. In addition, the 3% DMA concentration decreased the MDA levels while the activity of GPX and SOD antioxidant enzymes increased. Moreover, the 3% DMA concentration caused upregulation in the expression of some anti-freezing-related genes, which might enhance the frozen-thawed sperm characteristics. Thus, according to results obtained in the present study, the addition of 3% DMA in freezing extender preserves the post thaw sperm quality of chicken semen.

## Figures and Tables

**Table 1:** Primer sequences utilized in qRT-PCR analysis of designated anti-freeze-related genes.

Gene	Gene Full Name	Gene Bank Accessing Number	Primer Sequences (5′-3′3′)	Product Size
HSP70	heat shock protein 70	FJ217667.1	F-TTGATAAGGGCCAGATCCAG	105
			R-TGTTCAGCTCTTTGCCATTG	
RHOA	ras homolog family member A	NM_204704.1	F-GAAGCAGGAGCCTGTCAAAC	132
			R-GCAGCTCTAGTGGCCATTTC	
SNRPA1	small nuclear ribonucleoprotein polypeptide A	NM_001005823.1	F-CGACCTGCGGGGGTATAAAA	176
			R-GTCCTTCCCCAATCCGACAA	
RPL29	ribosomal protein L29	NM_001171677.1	F-GTCCCGTAAGTGGCACAGAA	157
			R-CTGCTTGGCATTGTTGGCTT	
GAPDH	glyceraldehyde-3-phosphate dehydrogenase	NM_204305.1	F-AGAACATCATCCCAGCGTCCA	130
			R-CAGGTCAGGTCAACAACAGAG	

**Table 2:** Impact of different DMA concentrations in semen freezing extender on the motion criteria of post-thawed rooster sperm.

	DMA Concentration			
Parameters	3%	6%	9%	*p*-Value
TMOT (%)	62.04 ± 0.882 ^a^	57.93 ± 0.784 ^ab^	54.73 ± 2.512 ^b^	0.019
PROG (%)	35.64 ± 0.839 ^a^	29.39 ± 1.209 ^b^	29.50 ± 1.140 ^b^	0.001
VAP (µm/s)	57.02 ± 0.979	55.24 ± 1.210	55.77 ± 1.900	0.667
VCL (µm/s)	103.38 ± 2.770	101.91 ± 2.900	104.90 ± 4.192	0.821
VSL (µm/s)	34.71 ± 0.414	34.90 ± 0.531	33.88 ± 0.964	0.541
STR (%)	60.70 ± 0.256	62.91 ± 0.833	60.64 ± 1.495	0.220
LIN (%)	33.49 ± 0.452	34.07 ± 0.925	32.46 ± 1.094	0.433
WOB (%)	54.84 ± 0.574	53.86 ± 0.904	52.89 ± 0.487	0.160
ALH (µm)	4.96 ± 0.083	4.84 ± 0.022	5.04 ± 0.063	0.098
BCF (Hz)	24.26 ± 0.346	23.63 ± 0.049	23.89 ± 0.667	0.601

Data are illustrated as means ± standard error (SE). Significantly (*p* < 0.05) different means within the same row are denoted by different superscripts. TMOT: total motility; PROG: progressive motility; VAP: average velocity line (µm/s); VCL: curved velocity line (µm/s); VSL: velocity straight line (µm/s); STR: straightness (VSL/VAP, %); LIN: linearity (VSL/VCL, %); WOB: wobble (VAP/VCL, %); ALH: amplitude of lateral head displacement (µm); BCF: beat cross frequency (H_z_).

**Table 3:** Impact of different DMA concentrations in semen freezing extender on the quality criteria of post-thawed rooster sperm.

	DMA Concentration			
Parameters	3%	6%	9%	*p*-Value
Viability (%)	75.05 ± 1.928 ^a^	71.18 ± 0.832 ^ab^	66.37 ± 1.145 ^b^	0.002
Intact acrosome (%)	89.45 ± 0.143 ^a^	91.04 ± 0.682 ^a^	84.51 ± 1.236 ^b^	<0.001
Intact plasma membrane (%)	90.65 ± 0.614 ^a^	87.37 ± 1.068 ^b^	83.83 ± 0.510 ^c^	<0.001

Data are illustrated as means ± standard error (SE). Significantly (*p* < 0.05) different means within the same row are denoted by different superscripts.

**Table 4:** Impact of different DMA concentrations in semen freezing extender on the antioxidant defense status of post-thawed rooster sperm.

	DMA Concentration			
Parameters	3%	6%	9%	*p*-Value
TAC (µM/mg)	0.013 ± 0.0032	0.020 ± 0.0040	0.014 ± 0.0021	0.253
GPX (mU/mg)	2.12 ± 0.365 ^a^	1.39 ± 0.099 ^ab^	0.66 ± 0.107 ^b^	0.002
SOD (U/mg)	23.57 ± 0.678 ^a^	15.92 ± 0.941 ^b^	14.52 ± 1.178 ^b^	<0.001
MDA (nM/mg)	0.054 ± 0.0039 ^c^	0.111 ± 0.0060 ^b^	0.144 ± 0.0114 ^a^	<0.001

Data are illustrated as means ± standard error (SE). Significantly (*p* < 0.05) different means within the same row are denoted by different superscripts. MDA: malondialdehyde, TAC: total antioxidant capacity, GPX: glutathione peroxidase, and SOD: superoxide dismutase. MDA, TAC, GPX, and SOD levels were normalized per mg protein.

## Data Availability

The data presented in this study are available on request from the corresponding author.
